# Nonalcoholic steatohepatitis and hepatocellular carcinoma: Brazilian survey

**DOI:** 10.6061/clinics/2016(05)07

**Published:** 2016-05

**Authors:** Helma P. Cotrim, Claudia P. Oliveira, Henrique Sérgio M. Coelho, Mario R. Alvares-da-Silva, Leticia Nabuco, Edison Roberto Parise, Claúdia Ivantes, Ana LC Martinelli, João Galizzi-Filho, Flair J. Carrilho

**Affiliations:** I Universidade Federal da Bahia, Departamento de Medicina – Serviço de Gastro-Hepatologia, Salvador/BA, Brazil; II Universidade de São Paulo, Departamento de Gastroenterologia, São Paulo/SP, Brazil; III Universidade Federal do Rio de Janeiro, Departamento de Clínica Médica, Rio de Janeiro/RJ, Brazil; IV Universidade Federal do Rio Grande do Sul, Hospital de Clínicas de Porto Alegre, Departamento de Medicina Interna, Serviço de Gastroenterologia, Porto Alegre/RS, Brazil; V Hospital Federal dos Servidores do Estado, Serviço de Clinica Médica, Setor de Hepatologia, Rio de Janeiro/RJ, Brazil; VI Hospital Nossa Senhora das Graças. Serviço de Gastroenterologia, Hepatologia e Transplante Hepático, Curitiba/PR, Brazil; VII Universidade de São Paulo, Faculdade de Medicina de Ribeirao Preto, Divisao de Gastroenterologia, Departamento de Clinica Medica, Ribeirão Preto/SP, Brazil; VIII Universidade Federal de Minas Gerais, Departamento de Clínica Médica, Belo Horizonte/MG, Brazil; IX Members of the NAFLD – HCC Survey - Sociedade Brasileira de Hepatologia (SBH), São Paulo/SP, Brazil

**Keywords:** Non-alcoholic Fatty Liver Disease, Nonalcoholic Steatohepatitis, Hepatocellular Carcinoma, Fatty Liver

## Abstract

**OBJECTIVE:**

The majority of cases of hepatocellular carcinoma have been reported in individuals with cirrhosis due to chronic viral hepatitis and alcoholism, but recently, the prevalence has become increasingly related to nonalcoholic steatohepatitis around the world. The study aimed to evaluate the clinical and histophatological characteristics of hepatocellular carcinoma in Brazilians' patients with nonalcoholic steatohepatitis at the present time.

**METHODS:**

Members of the Brazilian Society of Hepatology were invited to complete a survey regarding patients with hepatocellular carcinoma related to nonalcoholic steatohepatitis. Patients with a history of alcohol intake (>20 g/day) and other liver diseases were excluded. Hepatocellular carcinoma diagnosis was performed by liver biopsy or imaging methods according to the American Association for the Study of Liver Diseases' 2011 guidelines.

**RESULTS:**

The survey included 110 patients with a diagnosis of hepatocellular carcinoma and nonalcoholic fatty liver disease from nine hepatology units in six Brazilian states (Bahia, Minas Gerais, Rio de Janeiro, São Paulo, Paraná and Rio Grande do Sul). The mean age was 67±11 years old, and 65.5% were male. Obesity was observed in 52.7% of the cases; diabetes, in 73.6%; dyslipidemia, in 41.0%; arterial hypertension, in 60%; and metabolic syndrome, in 57.2%. Steatohepatitis without fibrosis was observed in 3.8% of cases; steatohepatitis with fibrosis (grades 1-3), in 27%; and cirrhosis, in 61.5%. Histological diagnosis of hepatocellular carcinoma was performed in 47.2% of the patients, with hepatocellular carcinoma without cirrhosis accounting for 7.7%. In total, 58 patients with cirrhosis had their diagnosis by ultrasound confirmed by computed tomography or magnetic resonance imaging. Of these, 55% had 1 nodule; 17%, 2 nodules; and 28%, ≥3 nodules.

**CONCLUSIONS:**

Nonalcoholic steatohepatitis is a relevant risk factor associated with hepatocellular carcinoma in patients with and without cirrhosis in Brazil. In this survey, hepatocellular carcinoma was observed in elevated numbers of patients with steatohepatitis without cirrhosis.

## INTRODUCTION

Hepatocellular carcinoma (HCC) is considered the 5th most common cancer in the world and it is responsible for 5% of all malignant tumors in humans 1. It is also the most frequent cause of all liver cancers, constituting 90% of cancers of the liver globally and it has been estimated to be 3rd most common cause of cancer-related death in humans 1-4.

The annual incidence of HCC among large series of Brazilian cirrhotic patients was approximately 2.9%, with a detection rate of 8.1% and a cumulative incidence rate over five years of 14.3% 5.

The majority of cases of HCC have been reported to be coincident with the presence of advanced chronic liver disease associated with chronic hepatitis B virus (HBV) or hepatitis C virus (HCV) infection or alcoholic cirrhosis.

Cryptogenic cirrhosis (CC) is observed in 15-30% of patients with advanced liver disease, and certain studies have suggested that nonalcoholic fatty liver disease (NAFLD) may be a risk factor for this condition 6,7. Moreover, the growing prevalence of HCC around the world may be related to NAFLD or nonalcoholic steatohepatitis (NASH).

In 2000, one of the first cases of HCC related to NASH was reported in Brazil 8, and recently, two other Brazilian studies on HCC and NAFLD were published 9,10. The present survey aimed to evaluate the clinical and histopathological characteristics of HCC in patients with NAFLD/NASH throughout the country at the present time.

## METHODOLOGY

### Study design and population selection

Members of the SBH (Brazilian Society of Hepatology) were invited to complete a survey regarding patients with HCC related to NAFLD/NASH. The questionnaire included questions about clinical and histological data from chart review and also prospective analysis.

HCC was diagnosed according to the noninvasive diagnostic criteria of the American Association for the Study of Liver Diseases (AASLD), proposed in 2005 and updated in 2011 11. Liver histology confirmation was performed in all non-cirrhotic patients and in inconclusive cases by imaging examination (computed tomography (CT) and/or magnetic resonance imaging (MRI)).

The criteria for NAFLD diagnosis included the presence of risk factors for NAFLD or metabolic syndrome, a history of ethanol intake of <20 g/day, and exclusion of other liver diseases (HBV and HCV infection, hemochromatosis, and autoimmune hepatitis).

The Ethics Committee on Human Research at Faculdade de Medicina da Bahia - Universidade Federal da Bahia (UFBA), Brazil, approved the study. This study was conducted according to the principles outlined in the Declaration of Helsinki (1964, revised 2008).

### Patient evaluation

Clinical evaluation included gender, age, anthropometric measures, blood pressure levels, history of type 2 diabetes, arterial hypertension, dyslipidemia, acute myocardial infarction, alcohol intake, smoking habits and use of medications (including anti-diabetic, lipid-lowering, antihypertensive and hepatotoxic drugs; glucocorticoids, estrogens, amiodarone, carbamazepine, and tamoxifen; and non-steroidal anti-inflammatory drugs).

Laboratory tests included assessment of total cholesterol (TC), high-density lipoprotein (HDL) cholesterol, low-density lipoprotein (LDL) cholesterol, triglycerides (TG), glucose, insulin, proteins, ferritin, transferrin saturation index, alanine aminotransferase (ALT), aspartate aminotransferase (AST), gamma-glutamyltransferase (GGT), alkaline phosphatase, hepatitis B surface antigen (HBsAg), anti-HCV and autoantibodies (anti-nuclear, anti-smooth muscle, and anti-mitochondria). Metabolic syndrome was defined according to the National Cholesterol Education Program Adult Treatment Panel III report 12.

### Statistical analysis

Descriptive analyses were conducted using Statistical Package for the Social Sciences software (SPSS Inc., Chicago, IL, USA; Release 16.0.2, 2008). The analyzed data are expressed as mean values, standard deviations, medians (Mdns) and interquartile ranges (IQRs) according to the variables' distribution.

## RESULTS

The survey included 110 patients with diagnoses of HCC and NAFLD from nine hepatology units in six Brazilian states (Bahia, Minas Gerais, Rio de Janeiro, São Paulo, Paraná and Rio Grande do Sul).

The mean age was 67±11 years old and 65.5% (72) were male. Obesity was observed in 52.7% (58) of cases; diabetes, in 73.6% (81); dyslipidemia, in 41.0% (45); arterial hypertension, in 60% (66); and metabolic syndrome, in 57.2% (63) (Table 1). Histological diagnosis of HCC was performed in 52 (47.2%) patients; among these 52 patients, NASH with cirrhosis was observed in 61.5%; NASH with fibrosis grades 1-3, in 27%; and NASH without fibrosis, in 3.8%. In 7.7% (4) of cases, the histological diagnosis was only HCC (Table 2). A total of 58 patients had a diagnosis of HCC by ultrasound confirmed by CT or MRI. Of these, 55% had 1 nodule; 17%, 2 nodules; and 28%, ≥3 nodules. All of these cases were associated with a cirrhotic liver (Table 3). Ascites was observed in 19.0% of 93 patients with HCC; portal hypertension, in 39.7% of 86 patients, hypoalbuminemia (<3 g/dL), in 8.6% of 93 patients; and a prothrombin time below 70%, in 21.0% of 83 patients.

## DISCUSSION

The present study suggests that HCC in Brazil is associated with NASH and is also observed in non-cirrhotic patients with and without fibrosis. Three previous studies performed in this country reported cases of HCC in patients with NASH 8-10. However, the present survey included a large number of patients from several Brazilian centers and regions.

NAFLD has been recognized as a frequent cause of HCC, especially in Western countries. Environmental factors, behavioral habits, metabolic features and inherited factors have been implicated in the pathogenesis of HCC 13.

CC is observed in 15-30% of patients with advanced liver disease and NASH has been associated with these cases 14.

Evidence that HCC is part of the natural history of NAFLD, which includes steatosis, NASH, and cirrhosis, comes from retrospective studies demonstrating that HCC is related to NAFLD risk factors, case reports, prospective studies that evaluated late complications in NAFLD patients and studies in animal models 15-18.

Certain aspects of the Brazilian survey described here deserve discussion. In particular, the most frequent risk factors found in patients with HCC and NASH were obesity and diabetes, metabolic syndrome was observed in 57.2% of these cases, and 72% of the cases presented fewer than three nodules and fulfilled the Milan inclusion criteria for liver transplantation. In this context, the major form of therapy for HCC would be liver transplantation. However, ascites was observed in 19.0% of 93 patients, and portal hypertension was observed in 39.7% of 86 patients, with approximately 30% presenting liver dysfunction. These findings suggest a late diagnosis of HCC in this survey, in which case it is difficult to administer curative treatment. Moreover, the main relevant finding in this study was the elevated number of HCC cases among NASH patients without cirrhosis. It was observed in 30.8% of the patients who underwent liver biopsy. Previously, researchers in our group identified HCC cases without cirrhosis in two published manuscripts 9,10. Several authors have also recently described this intriguing phenomenon of HCC in NASH patients without cirrhosis 17-19.

The physiopathology of the carcinogenesis of HCC in this liver disease is not clear. However, metabolic factors such as diabetes and obesity have been implicated.

In experimental studies with ob/ob rats (insulin-resistant and obese), HCC has been observed in the absence of cirrhosis 20. In these animals, the proliferation of hepatocytes was increased compared with apoptosis, suggesting that this imbalance could promote an increase in liver mass 21.

In humans, the physiopathology and molecular events that lead to HCC are not well understood. However, certain events have been observed: the proliferation of oval cells, which are progenitors of hepatocytes implicated as the origin of many liver tumors, including in patients with NAFLD 22; increased production of reactive oxygen species and oxidative injury 23,24; and mutations in regulatory genes, including tumor suppressors such as p53 and phosphatase tensin homolog (PTEN) 25.

The present study has certain limitations. For example, a survey is a type of study in which the information is descriptive. Therefore, it was not possible to follow up the patients; all of the information collected came from the charts of the patients, and it was not possible to obtain all of the clinical data. However, a survey can yield relevant results and it can also suggest points to be discussed. Based on the results of the present study, HCC in patients with NASH with or without cirrhosis seems to be a problem in Brazil, and this condition may become more frequent in the next few years. The results also suggest the relevance of the implementation of a surveillance protocol to investigate HCC in Brazilian NASH patients.

This survey suggests that NASH is a relevant risk factor for HCC in Brazil, whether associated with cirrhosis or not. In particular, HCC was observed in an elevated number of patients without cirrhosis. A surveillance protocol to investigate HCC in Brazilian NASH patients should be discussed.

## AUTHOR CONTRIBUTIONS

Cotrim HP served as project coordinator, wrote the project, contacted colleagues, sent files, organized the data, performed the analysis and wrote and organized the manuscript. Oliveira CP and Carrilho FJ sent patient files and helped to write and review the manuscript. Coelho SM, Nabuco L, Parise ER, Ivantes C, Martinelli AL and Filho JG sent patient files. Alvaresda-Silva MR sent patient files and helped to review the manuscript. Members of the SBH Brazilian HCC – NASH Survey sent patients files.

## Figures and Tables

**Table 1:** Baseline characteristics of the patients with nonalcoholic steatohepatitis and hepatocellular carcinoma (n=110).

	n	%
Gender		
Male	72	65.5
Female	38	35.5
Elevated aminotransferases	85	56.2
Metabolic syndrome	63	57.2
Obesity	58	52.7
Diabetes	81	73.6
Hypertension	66	60
Dyslipidemia	45	41

**Table 2:** Histological diagnosis of the patients with nonalcoholic steatohepatitis and hepatocellular carcinoma (n=52).

Diagnosis n	%
NASH + cirrhosis 32	61.5
NASH + fibrosis (grades 1-3) 14	27.0
NASH without fibrosis 2	3.8
HCC (clinical diagnosis 4 of NAFLD and cirrhosis)	7.7

**Table 3:** Imaging-based diagnosis of hepatocellular carcinoma (US* + CT** and/or MRI***) in the patients with nonalcoholic steatohepatitis (n=58).

Number of nodules	n	%
1	32	55
2	10	17
≥3	16	28
Portal hypertension	3	5.4

* Abdominal ultrasound

** Computed tomography

*** magnetic resonance imaging.
